# Effect of Ti Doping of Al_0.7_CoCrFeNi-Based High Entropy Alloys on Their Erosion Resistance by Solid Particles

**DOI:** 10.3390/ma18143328

**Published:** 2025-07-15

**Authors:** Wojciech J. Nowak, Tadeusz Kubaszek, Andrzej Gradzik, Małgorzata Grądzka-Dahlke, Dariusz Perkowski, Marzena Tokarewicz, Mariusz Walczak, Mirosław Szala

**Affiliations:** 1 Faculty of Mechanical Engineering and Aeronautics, Rzeszow University of Technology, Powstanców Warszawy 12, 35-959 Rzeszów, Poland; tkubaszek@prz.edu.pl (T.K.); andrzej_gradzik@prz.edu.pl (A.G.); 2 Faculty of Mechanical Engineering, Bialystok University of Technology, Wiejska 45 C, 15-351 Białystok, Poland; m.dahlke@pb.edu.pl (M.G.-D.); d.perkowski@pb.edu.pl (D.P.); marzena.tokarewicz@sd.pb.edu.pl (M.T.); 3 Faculty of Mechanical Engineering, Lublin University of Technology, Nadbystrzycka 36D, 20-618 Lublin, Poland; m.walczak@pollub.pl (M.W.); m.szala@pollub.pl (M.S.)

**Keywords:** solid particle erosion, high entropy alloys, hardness, Ti effect

## Abstract

The erosion resistance of materials against solid particles is a very important property, especially in the transportation of powders or in aeronautics (dust inside jet engines). There is a strong need to introduce new materials that have higher solid particle erosion resistance than state-of-the-art materials. Thus, in the present work, the solid erosion particles of high entropy alloys (HEAs) based on the Al0.7CoCrFeNi matrix were studied compared to the state-of-the-art stainless steel AISI 304. Furthermore, the effect of the addition of Ti to HEAs on hardness and erosion resistance was investigated. Current research included the development of the chemical composition of a new kind of HEA designed on the basis of thermodynamical calculations performed in CALPHAD, its manufacturing, full characterization involving microstructural and phase analyses, hardness measurements, solid particle erosion tests, and finally, the elucidation of erosion mechanisms. It was found that HEAs showed higher hardness as well as erosion resistance than AISI 304. Moreover, it was found that the increase in Ti content in an HEA resulted in an increase in the hardness and resistance to the erosion of the studied HEA. As the main reason for this phenomenon, the stabilization of the β-BCC phase, suppression of the α-FCC phase, and the appearance of the Ni_3_Ti phase in the studied HEA were claimed.

## 1. Introduction

Metallic materials are widely used in all sectors of industry where they face harsh conditions. Metal materials, especially steels, are often used in conditions that expose these materials to abrasive and erosive wear. This wear can occur as a result of various mechanisms. The materials used to manufacture cutting tools or friction joint elements wear out as a result of tribological interactions [1]. In turn, metallic materials used in aircraft engines can be destroyed by erosion at low and elevated temperatures [2]. An example is the suction of volcanic dust present in the air or sand dust that causes the erosion of the fan blades or blades in the compressor. Hence, there is a need to increase the resistance of alloys, e.g., by developing new alloys with desired properties such as resistance to erosion. The state-of-the-art materials used for anti-erosion coatings are projected in terms of their implementation, i.e., Stellite [3] is used in applications requiring high friction/erosion resistance. In high-temperature applications, MCrAlY-type coatings [4,5,6] or aluminide coatings [7,8] are used. However, Stellite, in its chemical composition, contains WC, which at a high temperature oxidizes very quickly, whereas MCrAlY coatings do not exhibit high wear resistance, and aluminide coatings are additionally very brittle. As mentioned above, these types of materials are used as coatings. This implements an additional cost. Therefore, in the present work, research on high entropy alloys (HEAs) as a candidate for an erosion-resistant material is proposed. HEAs are a relatively new sort of metallic material consisting of at least five major alloying elements. The first publications appeared not long ago [9,10]. HEAs are being considered for a wide range of functional and structural applications [11]. Various works have been carried out on the wear resistance of high-entropy alloys [12]. The effect of Ti addition to a Co-Cr_2.5_-Fe-Ni_2_ HEA was investigated by Gu et al. [13]. This in turn resulted in the decrease in the mass loss of the studied HEA. Zhang et al. showed that the relationship between BCC and FCC can also be controlled by the variation in Fe content in Al-Co-Cr-Fe_x_-Ni [14] and also in Fe_x_-Ni_2_-Co_2_-Cr-Ti-Nb [15]. The addition of Ti to Al_2_-Cr-Fe-Ni-Co-Cu-Ti_x_ caused an increase in the Laves phase content, which also altered the positive wear resistance of the alloy studied. Gu et al. [16] showed that an increase in Al content leads to the transformation from a single BCC phase to a two-phase BCC in an Alx-Mo_0.5_-Nb-Fe-Ti-Mn_2_ HEA. In both works, the authors found that variation in element content influenced wear resistance. It is also known that Ni increases resistance to electrochemical corrosion. Research on the influence of Ni addition in Al_2_-Cr-Fe-Co-Cu-Ti-Ni_x_ HEA coatings was carried out by Qiu et al. [17]. The addition of Ni increased the hardness of the coatings; however, the addition above 1% caused the brittleness of the coatings and, in turn, worsened its wear resistance. In a recent study, Tokarewicz et al. [18] found that alloying with the V of an Al_0.7_CoCrFeNi HEA increased the wear resistance of the systems studied. In terms of solid particle erosion resistance, medium entropy alloys were investigated. Xu et al. [19] studied the effect of Y addition to an AlCrFeNi MEA. They found that increasing the addition of Y causes higher solid particle erosion of the studied MEA. Solid particle erosion of the HEA was also investigated. Wang et al. [20] studied the erosion resistance of a dual-phase AlCoFeNi_2_ HEA. The results revealed that the studied HEA exhibited an erosion rate lower by 10% than 316 SS. Research conducted on HEA-made coatings showed their lower erosion rate than conventionally used materials [21,22,23,24]. An AlCoCrFeNi-type HEA has already been studied in the literature. The results showed that the type of structure of the Al_x_CoCrFeNi alloy depends on the aluminum content. The increase in Al contributes to the change in the FCC crystal structure to BCC while reducing the energy of lattice distortion. Cr and Fe are responsible for stabilizing the BCC phase in the alloy, while Ni and Co stabilize the FCC phase [18,25,26]. Increasing the Al content causes an increase in hardness and strength while decreasing the plasticity of the alloy. Too high level of elements, such as aluminum and chromium, promotes the formation of intermetallic compound phases in the alloy, causing its brittleness. The Al_0.7_CoCrFeNi alloy belongs to the two-phase (FCC + BCC) high entropy alloy, which is a mixture of ductile and hard phases. This allows for maintaining a good balance between the strength and plasticity of the material. According to reports in the literature, the alloy is produced by arc melting in a vacuum, and directional crystallization is characterized by a lamellar-dendritic structure. Such a structure is formed by the disordered FCC and BCC phases. The lamellar structure is formed by the precipitation of the BCC phase from the FCC dendrite during the cooling process. The interdendritic structure is composed of the B2 phases and the disordered BCC phase [27]. The wear mechanism of the Al_0.7_CoCrFeNi alloy is of an adhesive–abrasive nature, during which a secondary layer is formed, which does not protect the material during further wear [18,28]. The effect of Ti on a CoCrFeNiCu_1-y_Al_y_ HEA has already been conducted by several authors, for example, Zhang et al. [29]. The alloying by Ti of Al0.7CoCrFeNi was already also studied. The AlCoCrFeNiTi_x_ alloy is usually characterized by a two-phase structure consisting of the FCC and BCC phases. The final crystal structure of the alloy is related not only to the amount of aluminum but also to titanium, which, after exceeding a certain content, stabilizes the BCC phase. Alloys with a titanium content of about 0.5 wt.% may have a structure containing two BCC phases. One of them is formed by an ordered BCC phase rich in Al and Ni, while the other is a disordered BCC phase rich in iron and chromium. The addition of titanium in the alloy may lead to the formation of interdendrites characterized by a honeycomb structure. Titanium contained in the alloy increases its strength and hardness by strengthening the solid solution, leading to increased resistance to abrasive wear [25,30]. It was also found that Ti also influences the mechanical properties of an AlCoCrFeNi HEA. It was found that the addition of 0.5 mole of Ti to the studied HEA resulted in the best mechanical properties. An increased amount of Ti added caused the worsening of alloy mechanical properties of the alloy [31]. All the mentioned research indicated a huge potential for HEAs as erosion-resistant materials. Therefore, in the present work, a systematic investigation of solid particle erosion tests of the HEA Al_0.7_CoCrFeNiTi_x_, where x=0, 0.05, 0.2, and 0.5 mole, was performed. The results obtained were compared to the state-of-the-art material, which is stainless steel AISI 304.

## 2. Materials and Methods

In the present work, two types of materials, namely high entropy alloys based on Al_0.7_CoCrFeNiTi_x_, where x is 0, 0.05, 0.2, and 0.5 mole, and commercially available stainless steel AISI 304 were investigated in terms of their resistance to erosion by solid particles. In the text, stainless steel will be called AISI 304, Al_0.7_CoCrFeNiTi_0_—Ref., Al_0.7_CoCrFeNiTi_0.05_—Ref.+0.05Ti, Al_0.7_CoCrFeNiTi_0.2_—Ref.+0.2Ti, and Al_0.7_CoCrFeNiTi_0.5_—Ref.+0.5Ti. The chemical composition of the stainless steel AISI 304 is given in Table 1, while the projected and obtained chemical composition of the HEA is shown in Table 2 and Table 3, respectively. The chemical composition of the studied HEA was designed on the basis of thermodynamical calculations performed in CALPHAD.

AISI 304 was delivered in the form of a rod with 30mm diameter. The HEAs were manufactured from high-purity powders (purity greater than 99.8%). The procedure was as follows. In the first step, the powders were balanced in proper proportions to obtain the projected chemical composition of the bulk HEA and mixed in ball mills for 2 h to obtain a uniform distribution of the alloying elements. After ball milling, the powders were pressed into 20 mm diameter and 10 mm height pellets using a hydraulic press. These prepared pellets were placed in an Arc-Melter (Edmund Buehler, Tubingen, Germany) and a melting process was performed. The melting chamber was pumped up to 5 × 10^−2^ mbar and was subsequently flushed with high-purity argon (purity 5N). This flushing process was repeated 3 times. After the third time, a high vacuum (5 × 10^−5^ mbar) was produced using a diffusion pump. After that, a small amount of Ar was introduced into the melting chamber, and the arc melting process was performed. After melting each sample, they were turned around 180°. This procedure was repeated 5 times to ensure a uniform chemical composition throughout the sample volume. After the melting process, the samples were cooled with the furnace to room temperature and taken out. From each kind of studied material, samples in a rectangular shape of 20 × 20 × 5 mm were cut. The surfaces of the samples were ground to 1200 grit sandpaper. Such prepared samples were subjected to an erosion test by solid particles using an Air Jet Erosion Tester TR-470 (Koehler, Germany) according to the norm G76. Measurement conditions were established as follows: temperature, 25 °C; duration, 10 min; powder flow, 2 g/min; work gas pressure, 19.6 Pa. Erosion tests were performed under 30° and 90°. The mass change was measured using the KERN ABT 120-4M (KERN & SOHN GmbH, Balingen, Germany) with a precision of 1 × 10^−4^ g. Phase analysis was conducted by X-ray diffraction (XRD) using an X-ray diffractometer Miniflex II made by Rigaku (Tokyo, Japan). The copper lamp (CuKα, λ = 0.1542 nm) with a voltage of 40 kV was used as an X-ray source. The measured 2θ angle ranged between 20 and 100° with a step size of 0.02°/s. The microstructure of the materials under cast conditions was investigated on cross sections and prepared according to standard metallographic procedures using the Hitashi S3400N scanning electron microscope (Hitashi, Tokyo, Japan). Elemental mapping of materials under as-cast conditions and also the surface of samples after erosion tests were performed using the scanning electron microscope Phenom XL (Thermo Fisher Scientific Inc., Waltham, MA, USA). The powder analysis of the erosion agent was performed using a laser diffraction method on an IPS U particle size analyzer (Kamika Instruments Warsaw, Poland). The craters originating from erosion tests were analyzed using a Sensofar S-Neox Non-contact 3D optical profiler laser profilometer (Sensofar, Barcelona, Spain).

## 3. Results

The present work consists of studies performed on two types of materials, namely stainless steel AISI 304 (representative of the state-of-the-art materials) and high entropy alloys consisting of Ref.+Ti, where x is 0, 0.05, 0.2, and 0.5 moles. The studied HEAs are analyzed and proposed as a material possessing better resistance for solid particle erosion tests than state-of-the-art materials.

### 3.1. Characterization of Materials in the As-Cast Conditions

**Figure 1:**
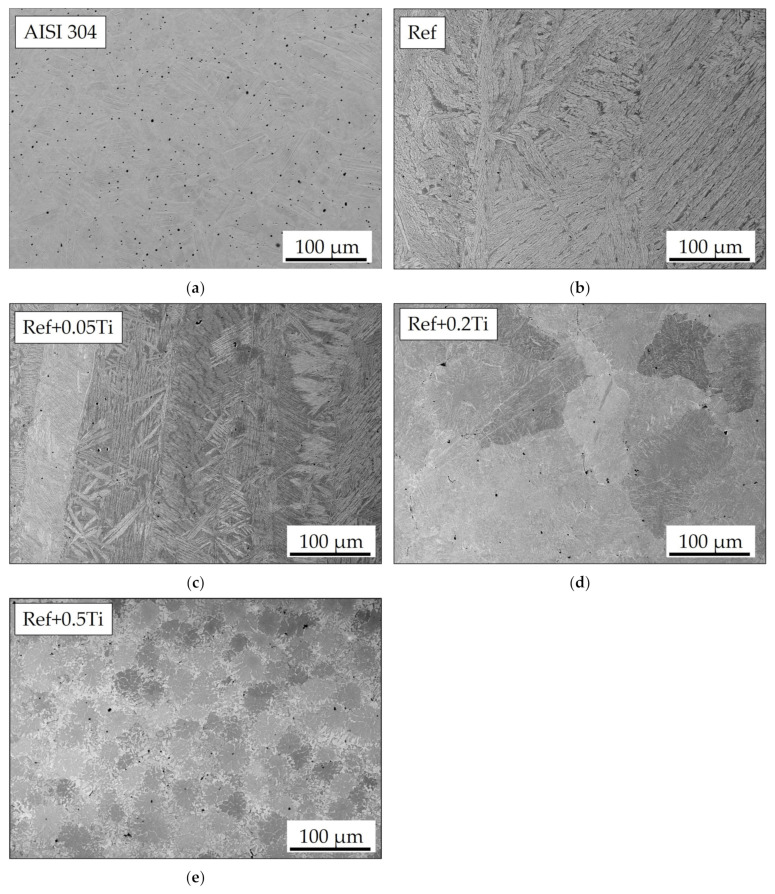
SEM/BSE images showing microstructure of (**a**) AISI 304, (**b**) Ref., (**c**) Ref.+0.05Ti, (**d**) Ref.+0.2Ti, and (**e**) Ref.+0.5Ti in the as-cast conditions taken at low magnification.

**Figure 2:**
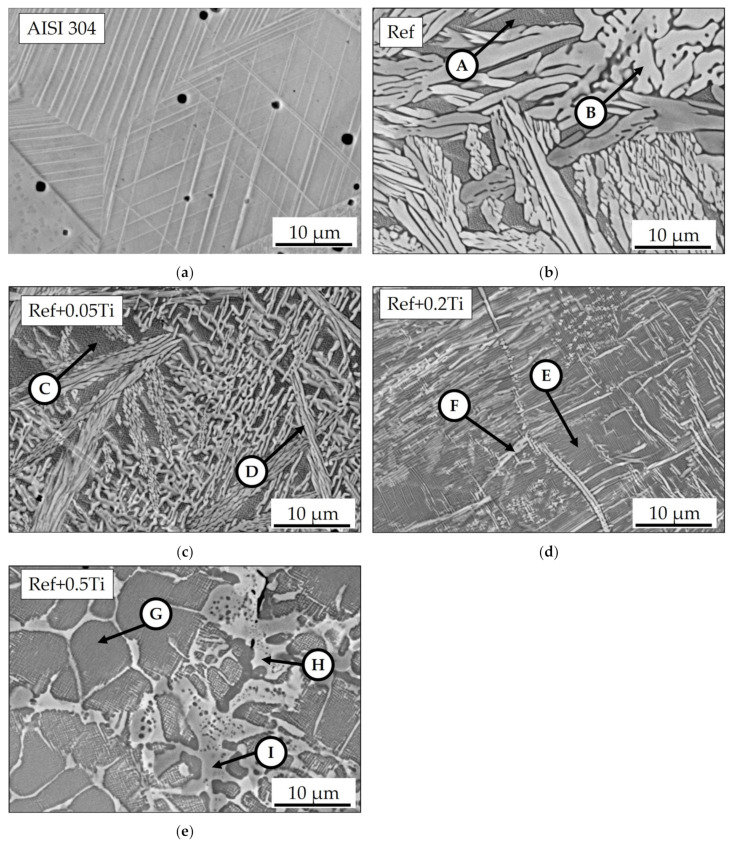
SEM/BSE images showing microstructure of (**a**) AISI 304, (**b**) Ref., (**c**) Ref.+0.05Ti, (**d**) Ref.+0.2Ti, and (**e**) Ref.+0.5Ti in the as-cast conditions taken at high magnification. Points indicate analysis spots. The results are shown in Table 4.

The microstructure of the studied materials under as-cast conditions is shown in Figure 1 at a lower magnification and Figure 2 at a higher magnification. The surface of AISI 304 was electrochemically etched to reveal its microstructure. The side effect of etching is the formation of voids that are present as dark dots shown in Figure 1a. Microstructural investigation of AISI 304 revealed that it shows a uniform microstructure over which a number of twins are present. The presence of twins is especially visible in Figure 2a. In the case of Ref., the HEA lamellar-dendritic structure was found (Figure 1b and Figure 2b). For Ref.+0.05 Ti, a thinning of the dendrite branches was observed (Figure 1c and Figure 2c). In the microstructure of Ref.+0.2Ti, further sectioning of dendrites and grain formation are also observed (Figure 1d and Figure 2d). Moreover, within a grain volume, some kind of mosaic structures are observed. For Ref. +0.5Ti, further grain refinement is observed and some kind of mosaic structure is observed (Figure 1e and Figure 2e).

**Figure 3:**
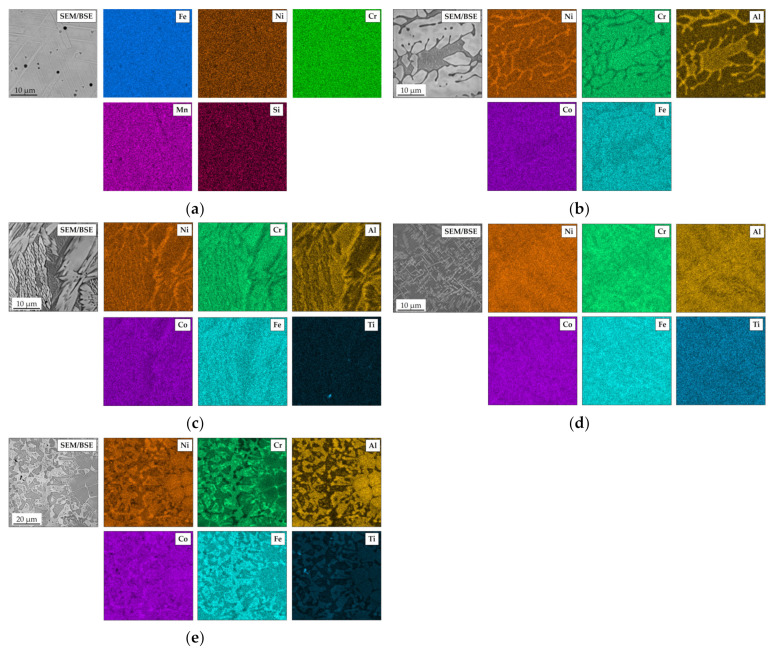
SEM/BSE elemental maps of (**a**) AISI 304, (**b**) Ref., (**c**) Ref.+0.05Ti, (**d**) Ref.+0.2Ti, and (**e**) Ref.+0.5Ti in as-cast conditions taken at high magnification.

The elemental SEM maps obtained for AISI 304 (Figure 3a) showed a uniform distribution of each measured element. For all HEAs, clear segregation of the elements is found. In Ref., the HEA in lighter regions of the SEM/BSE images for Cr, Co, and Fe are enriched, while in the darker region, mainly Al is enriched. At the edges of the Al-rich phases, Ni is also enriched (Figure 3b). A similar situation is found for Ref.+0.05Ti (Figure 3c). Ti seems to be coenriched in the same phase as Al. This phenomenon is found also for Ref.+0.2Ti (Figure 3d) and especially for Ref.+0.5Ti (Figure 3e). For a better illustration of the non-even distribution of alloying elements in the studied HEA, especially Ti, the locations of the SEM/EDS measurement points of the proper microstructural components are shown in Figure 2b–e. The results of the respective SEM/EDS measurements are given in Table 4. The dark gray phases (points A, C, E, G) are enriched in Al, Ti, and Ni, whereas the light gray phases (points B, D, F, H) are rich in Fe and Cr.

**Figure 4:**
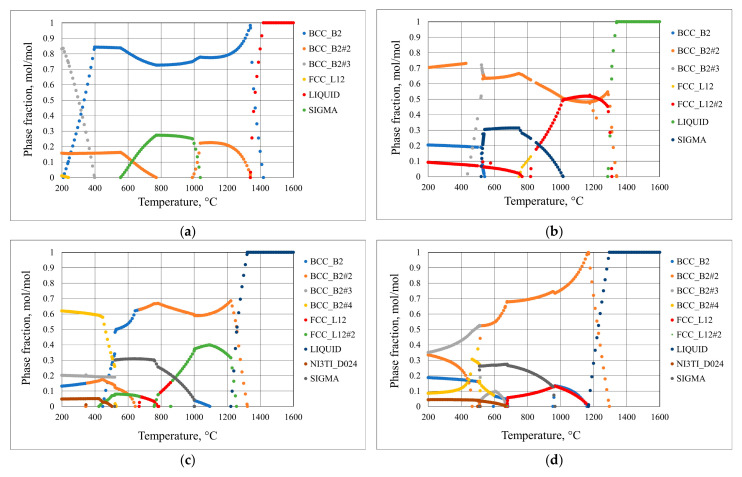
Plots showing the molar fraction of phases at a given temperature calculated using ThermoCalc software for (**a**) Ref., (**b**) Ref.+0.05Ti, (**c**) Ref.+0.2Ti, and (**d**) Ref.+0.5Ti.

On the basis of projected chemical composition, the phase composition was calculated in ThermoCalc software using TCHEA: TCS High Entropy Alloys Database, Perpetual DSUNI 7. The results of phase modeling for the investigated HEAs are shown in Figure 4a–d. As shown in Figure 4a near room temperature the Ref. consists of the BCC_B2#3 phase (0.83 mol/mol), BCC_B2#2 (0.16 mol/mol), and FCC_L12 (0.01 mol/mol). For Ref.+0.05Ti, the BCC_B2#2 phase is present (0.71 mol/mol) and so is BCC_B2 (0.20 mol/mol) and FCC_L12#2 (0.09 mol/mol). In the case of Ref.+0.2Ti, the FCC phase disappeared, while the following phases are found: BCC_B2#4 0.62 mol/mol), BCC_B2#3 (0.20 mol/mol), BCC-B2 (0.13 mol/mol), and the Ni_3_Ti phase occurred in the amount of 0.05 mol/mol. Finally, the calculated phase composition of Ref.+0.5Ti revealed the presence of BCC_B2#3 (0.35 mol/mol), BCC_B2#2 (0.33 mol/mol), BCC_B2 (0.18 mol/mol), BCC_B2#4 (0.09 mol/mol), and Ni_3_Ti (0.05 mol/mol). Thus, increasing the addition of Ti causes the suppression of the FCC phase and the occurrence of the Ni_3_Ti phase in the calculated phase composition of the studied materials.

**Figure 5:**
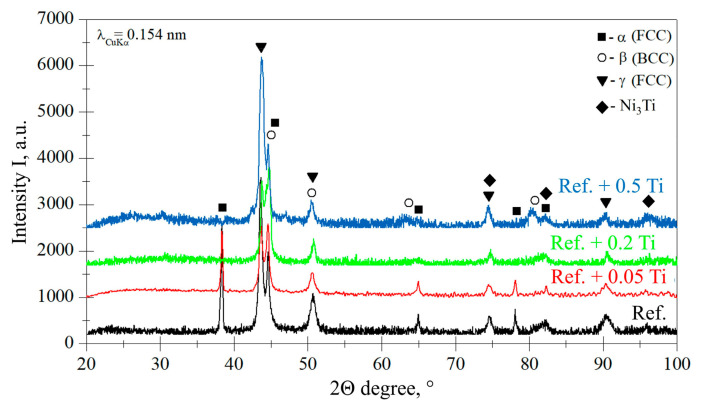
XRD diffraction patterns obtained for HEA studied under as-cast conditions.

The results of the phase analysis performed by XRD are shown in Figure 5. For Ref. and Ref. + 0.05Ti, the presence of α-FCC, β-BCC, and γ-FCC phases is found. Such a phase composition is observed also for alloy Ref.+0.05Ti. However, for an HEA with a higher Ti concentration, that is, for Ref.+0.2Ti and Ref. + 0.5Ti, peaks from α-FCC were not found for degrees 38, 65, and 78 (Figure 5). Additionally, peaks that show the presence of Ni_3_Ti phases are identified, especially for the alloy Ref.+0.5Ti (Figure 4), with peaks at 74.4, 82.3, and especially 96.3°. A fairly strong peak of the Ni_3_Ti phase is found for Ref.+0.5Ti, especially for 96.3° (Figure 4).

**Figure 6:**
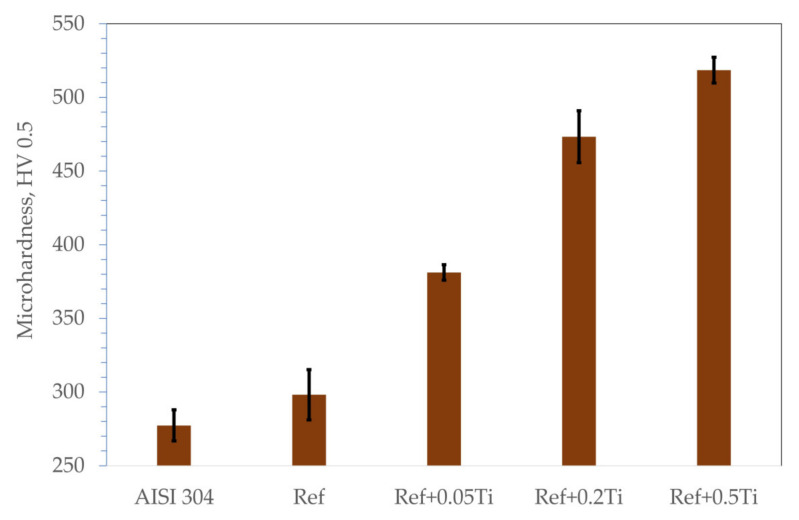
Microhardness expressed in HV0.5 obtained for the HEA studied under as-cast conditions.

Figure 6 shows the results of the microhardness measurement obtained on the materials studied. The results showed that AISI 304 revealed the smallest microhardness at a level of 280 HV0.5. Each studied HEA showed higher microhardness compared with AISI 304. Ref. HEA showed a microhardness of 300 HV0.5, Ref.+0.05Ti showed a microhardness of 380 HV0.5, Ref.+0.2Ti showed a microhardness of 480 HV0.5, and finally, Ref.+0.5Ti showed a microhardness of 570 HV0.5. Thus, an increase in the Ti content causes a significant increase in the alloy hardness.

### 3.2. Solid Particle Erosion Tests

**Figure 7:**
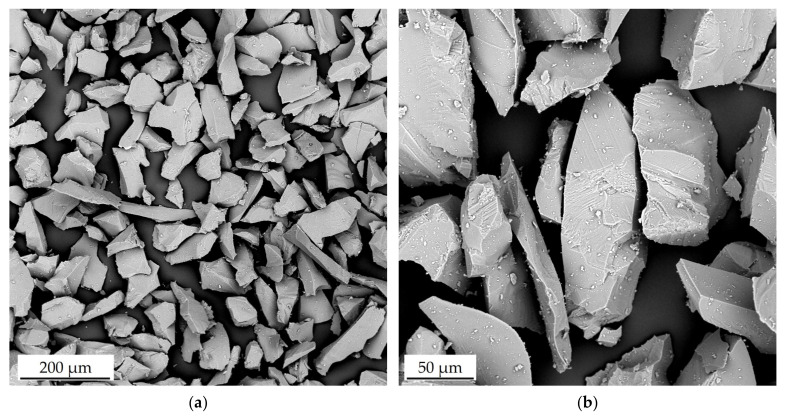
SEM/BSE images showing the morphology of powder used as erosive agent at (**a**) low and (**b**) high magnification.

**Figure 8:**
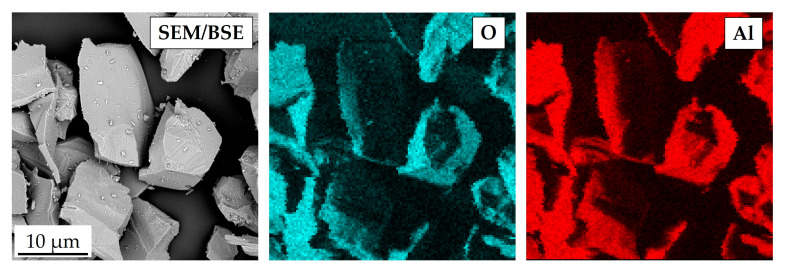
SEM/EDS elemental map obtained in the erosive agent used for the solid particle erosion test.

**Figure 9:**
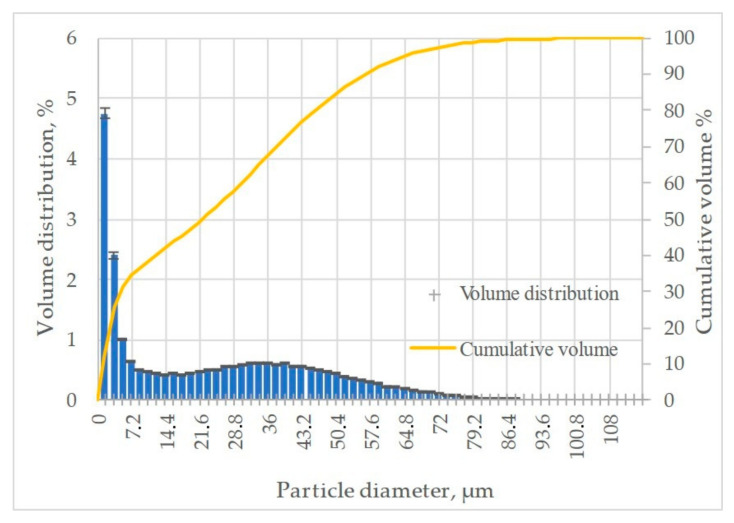
Plot showing the results of the powder analysis on the erosive agent.

Solid particle erosion tests were performed using the Air Jet Erosion Tester. As an erosive agent, crushed alumina particles were used. Figure 6 shows the morphology of the alumina powder at low magnification (Figure 7a) and high magnification (Figure 7b). It is visible that the powder particles possess sharp edges and parts of the grains are elongated. The elemental SEM map shown in Figure 8 confirms that the powder used is pure alumina. The results of the powder analysis are shown in Figure 9. Based on these results, the following powder dimensions were calculated: d_10_ = 1.54 µm, d_50_ = 22.15 µm, and d_90_ = 56.35 µm.

**Figure 10:**
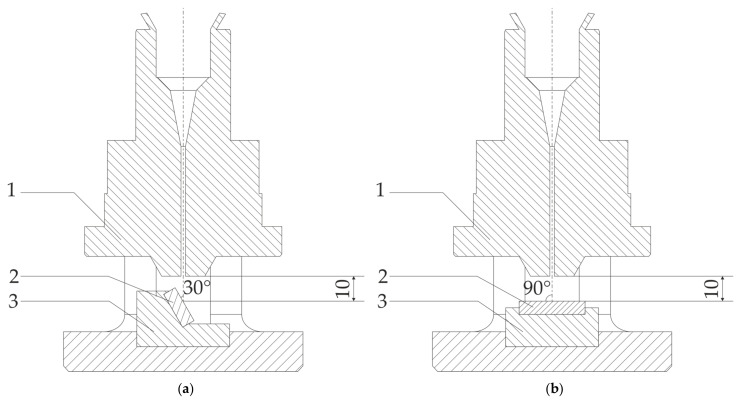
Schematic illustration of the equipment set up to perform solid particle erosion tests under (**a**) 30° and (**b**) 90°: 1, nozzle; 2, sample itself; and 3, sample holders.

**Figure 11:**
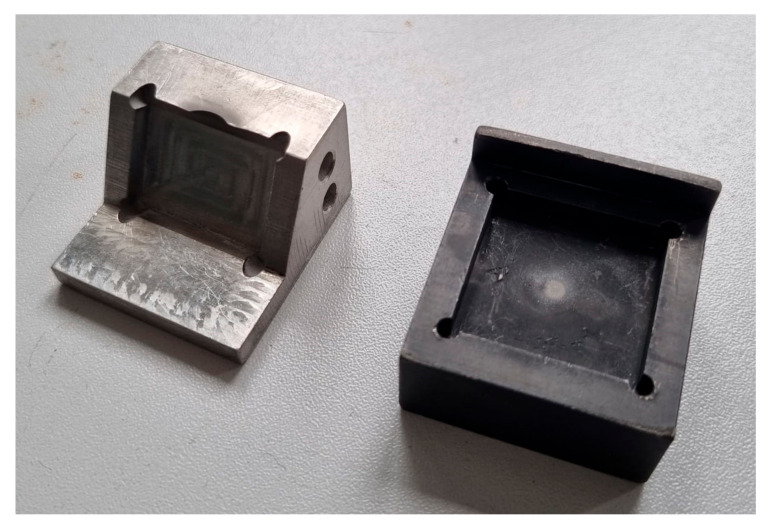
Images of sample holder used for solid particle erosion tests under 30° (**left-hand side**) and 90° (**right-hand side**).

**Figure 12:**
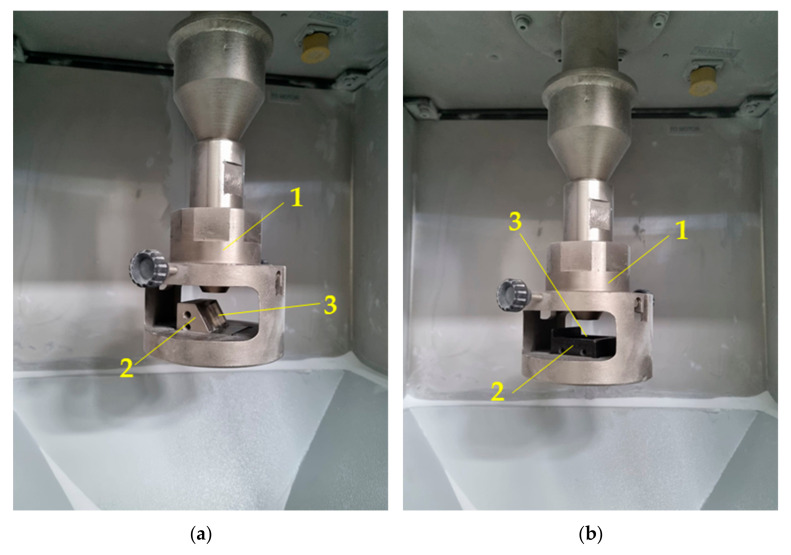
Images of equipment for solid particle erosion test with fixed sample holder for tests under (**a**) 30° and (**b**) 90°; 1, nozzle; 2, sample holder; and 3, place of fixing the sample.

The solid particle erosion tests were carried out under two different angles: 30° and 90°, as shown in Figure 10. In the case of the test performed at an angle of 30°, a stream of erosive particles emerging from the nozzle hits the surface of the studied sample at an angle of 30°, as shown in Figure 10a, while for the erosion test at an angle of 90°, the angle between the stream of erosive particles is 90° (Figure 10b). Figure 11 shows the sample holders used for the erosion test under 30° (left-hand side of Figure 11 and 90° (right-hand side of Figure 11). Figure 12 shows the equipment for the solid particle erosion test with mounted sample holders under 30° (Figure 12a) and 90° (Figure 12b). The sample holders are projected in such a manner to ensure that the distance between the nozzle and the sample surface is equal to 10 mm, as stated in the ASTM G76. After each test, the mass of the samples was measured using microbalance with an accuracy of 1 × 10^−4^ g.

**Figure 13:**
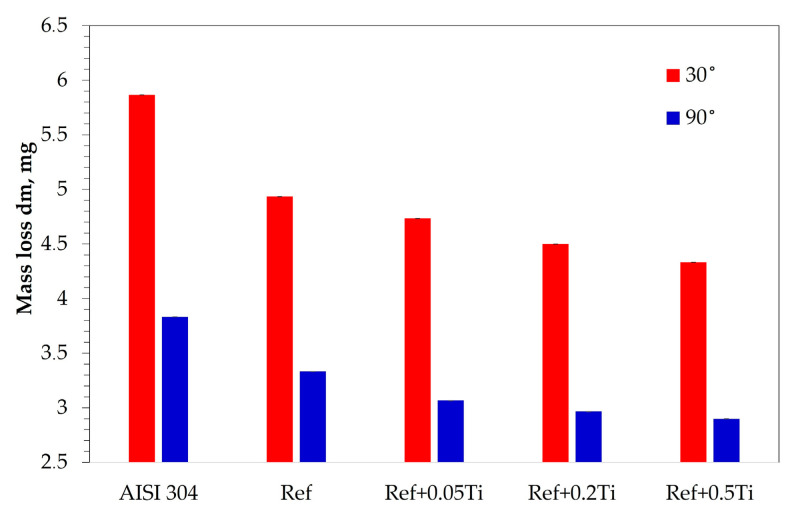
Plot showing the mass loss obtained during the solid particle erosion tests at different angles.

**Figure 14:**
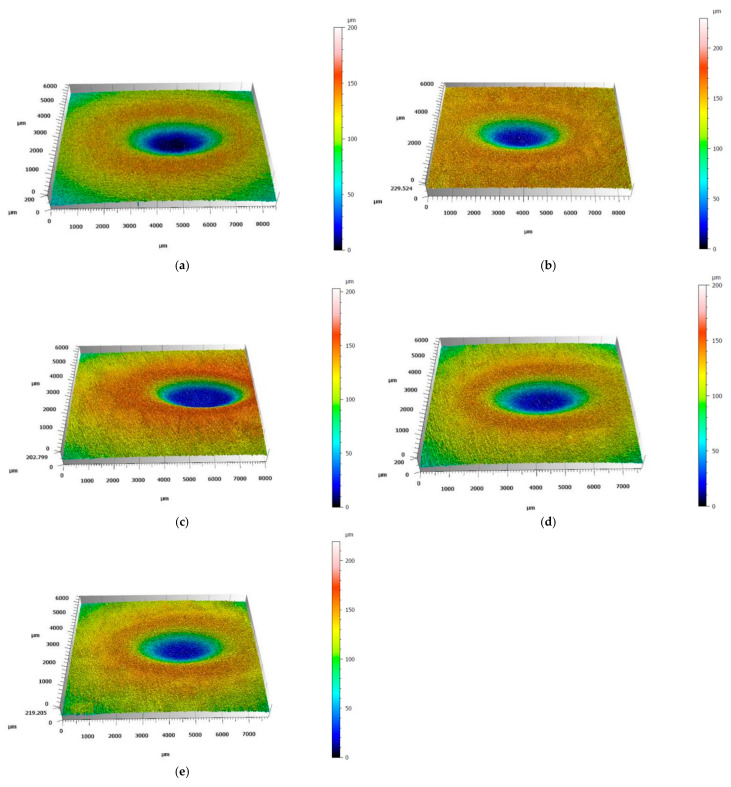
Images of craters formed during solid particle erosion tests at an angle of 30° in (**a**) AISI 304, (**b**) Ref., (**c**) Ref.+0.05Ti, (**d**) Ref.+0.2Ti, and (**e**) Ref.+0.5Ti obtained by laser profilometry.

**Figure 15:**
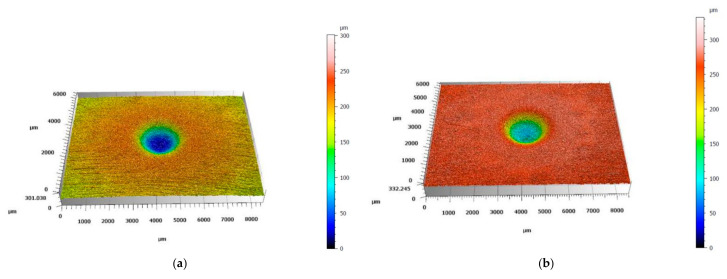

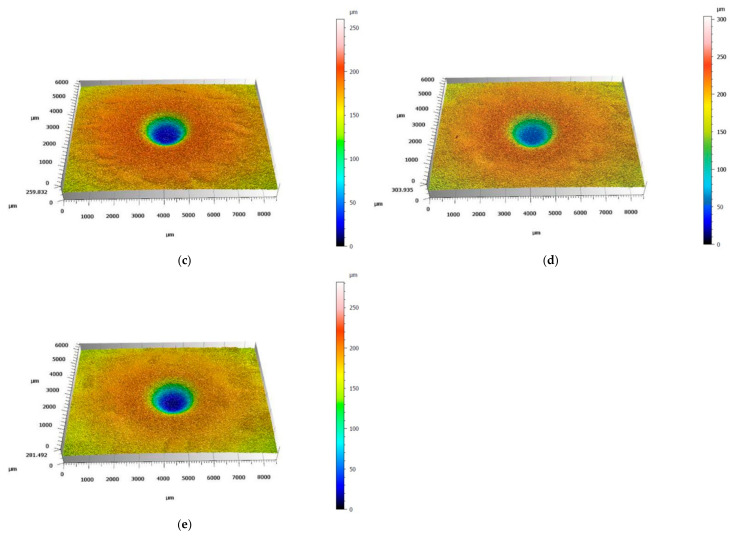
Images of craters formed during solid particle erosion tests under 90° on (**a**) AISI 304, (**b**) Ref., (**c**) Ref.+0.05Ti, (**d**) Ref.+0.2Ti, and (**e**) Ref.+0.5Ti obtained by laser profilometry.

The results of solid particle erosion tests are shown in Figure 13. In both tests, AISI 304 showed the highest mass loss. The results obtained for the studied HEA showed that each HEA revealed a smaller mass loss compared to AISI 304. Moreover, an increase in Ti content caused a decrease in mass loss. Furthermore, the values obtained for tests performed under 30° are higher than for 90° for each studied material. Figure 14 and Figure 15 show the images of the craters formed after erosion tests on the studied materials obtained by laser profilometry. Craters formed after the solid particle erosion test performed under 30° are larger in size and elongated, and they form a shape similar to an ellipse (Figure 14a–e). The craters formed after the erosion test at 90° are round (Figure 15a–e). In both cases, the craters are similar in shape; however, the depth of the craters becomes smaller starting from the crater on AISI 304 (Figure 14a and Figure 15a), for which the depths of the craters are the biggest, ending on Ref.+0.5Ti, for which the depths of the craters are the smallest one (Figure 14e and Figure 15e).

## 4. Discussion

Characterization of the materials studied in the as-cast condition showed that AISI 304 reveals the formation of one phase. The presence of relatively large numbers of twins was found (Figure 1a). This is the consequence of material preparation, that is, samples from AISI 304 were prepared from the rod with a 30 mm diameter. Thus, the material was extruded and plastically deformed during the manufacturing process. The latter caused the introduction of twins. For HEAs, it was found that in the case of the reference, HEA (Ref.) is characterized by a lamellar-dendritic structure. Such a structure is formed by the disordered FCC and BCC phases. The lamellar structure is formed by the precipitation of the BCC phase from the FCC dendrite during the cooling process. The interdendritic structure is composed of the B2 phases and the disordered BCC phase [27]. Increasing Ti content leads to the refinement of the microstructure for Ref.+0.05Ti, the formation of grains in the case of Ref.+0.2Ti (Figure 1d), and further refinement of the grains in the case of Ref.+0.5Ti (Figure 1e). For Ref.+0.2Ti and Ref. + 0.5Ti, the formation of mosaic microstructures within the grains is found. These mosaic structures are similar to Widmannstetter structures [32]. As shown by thermodynamic calculations, the addition of 0.05Ti caused an increase in the amount of the BCC_B2#2 phase. For medium entropy, an increase in yield strength was found by a factor of two by the occurrence of the BCC phase in the alloy [33]. Solid solution strengthening by Ti addition was claimed. Thus, a similar effect can be responsible for the hardness increase in the present work. Furthermore, the increase in Ti content caused the suppression of the FCC phase and the occurrence of the Ni_3_Ti phase for the HEA containing 0.2Ti and 0.5Ti. Ni3Ti is an intermetallic phase showing a higher hardness than the surrounding matrix [34]. In the present work, Ni_3_Ti precipitates were not found. However, thermodynamical calculations predicted the presence of small amounts of the Ni_3_Ti phase. XRD phase analysis revealed the presence of such a phase, especially for Ref.+0.5Ti. Therefore, considering the relatively high cooling rate of the studied materials during the arc-melting process, which can reach even 1 × 10^2^ K/s [35], the presence of an ultrafine Ni_3_Ti phase is possible. Therefore, the presence of Ni_3_Ti phases even in a small amount and in the form of ultrafine precipitates can contribute to an increase in hardness by precipitation hardening. It is known from the literature that a general increase in the hardness of the materials results in the resistance to a decrease in erosion [36,37]. This study shows that this is not always true. In this case, the increase in microhardness is proportional to the increase in erosion resistance. The erosion was caused by alumina (Al_2_O_3_), which was carried by pressurized air at an ambient temperature. Two different impingement angles were investigated, i.e., 30° and 90°, to comparatively analyze the erosion mechanisms of the HEA, type Ref.+Ti_x_, and the test conditions followed the ASTM-G76 standard requirements [38]. The quantitative results (Figure 13) indicate that in relation to the AISI 304 austenitic stainless steel, the HEAs show superior erosion resistance for both 30° and 90° impingement angles, as depicted in Figure 13, primarily attributed to their multiphase microstructures and high hardness, as shown in Figure 6, exceeding the hardness of the reference austenitic steel (approximately 230 HV). The high resistance to the erosion of different types of HEAs is attributed in the scientific literature [39,40,41] mainly to the increasing hardness.

**Figure 16:**
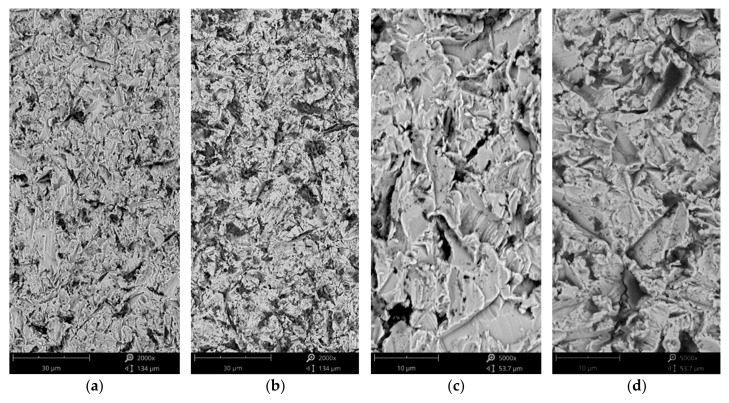
SEM/BSE images of eroded surfaces of AISI 304 stainless steel after erosion test under (**a**,**c**) 30° and (**b**,**d**) 90° shown at magnification of (**a**,**b**) ×2000 and (**c**,**d**) ×5000.

The test samples differ in microstructure and hardness, which are considered the most crucial factors determining the erosive behavior of metallic materials [42]. The austenitic steel AISI 304 erosion mechanism is comparable to those reported in the literature [43,44]. Representative eroded surfaces of stainless steel are presented in Figure 3. Due to dominant microploughing and the lesser action of microcutting, severe surface damage is shown for 30 degrees. This microploughing provides systematic plastic deformation and lip formation and further their removal by the impingement particles results in material detachment. The 90° test results in the extrusion of the material at the end of impact crates, fatigue of the surface, formation of grooves and lips, severe plastic deformation, and the further removal of materials. Furthermore, on the surface of the stainless steel (Figure 16), the stuck alumina particles are richer than those for the 30° test, which is a typical phenomenon observed in erosive testing [44,45].

**Figure 17:**
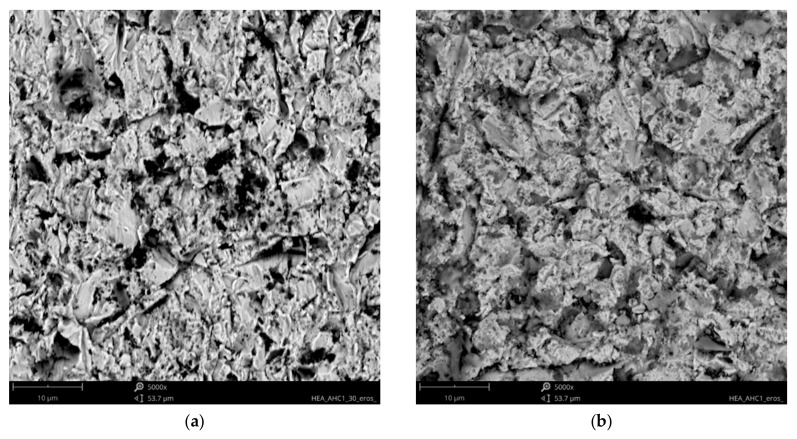
SEM/BSE images of eroded surfaces of Ref. HEA after erosion test under (**a**) 30° and (**b**) 90° shown at magnification of ×5000.

**Figure 18:**
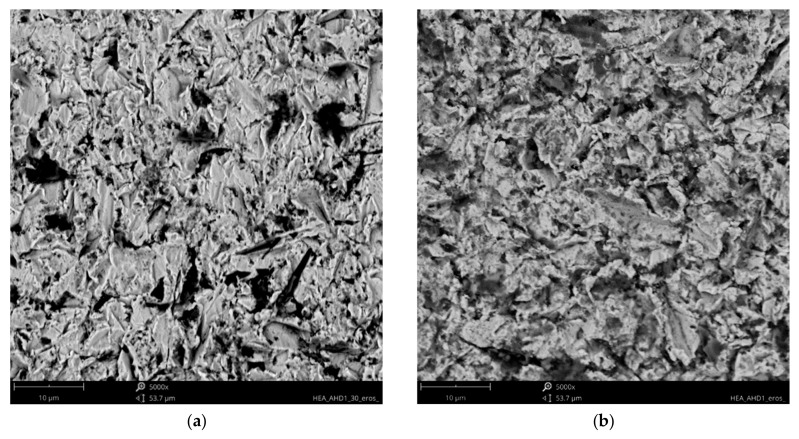
SEM/BSE images of eroded surfaces of Ref.+0.05Ti HEA after erosion test under (**a**) 30° and (**b**) 90° shown at a magnification of ×5000.

**Figure 19:**
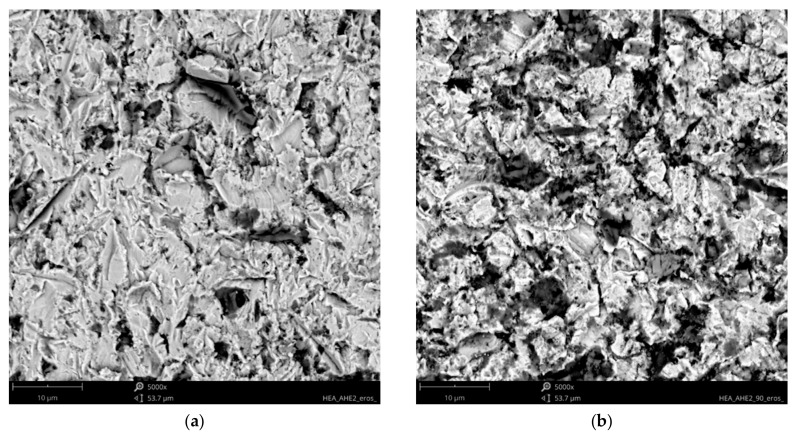
SEM/BSE images of eroded surfaces of Ref.+0.2Ti HEA after erosion test under (**a**) 30° and (**b**) 90° shown at a magnification of ×5000.

**Figure 20:**
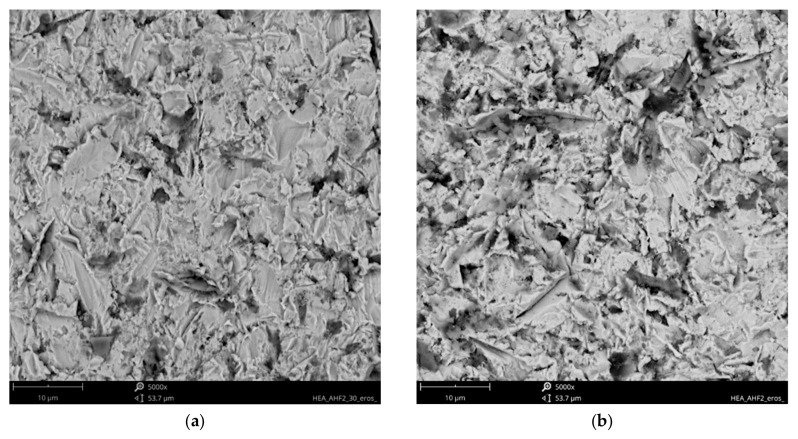
SEM/BSE images of eroded surfaces of Ref.+0.5Ti HEA after erosion test under (**a**) 30° and (**b**) 90° shown at magnification of ×5000.

Analysis of the available literature [39,40,41,46] indicates that the erosion mechanism of Ref.+Ti, where x = 0.05, 0.2, and 0.5 Ti, has not been studied sufficiently. The effect of titanium addition on erosion resistance has not been clarified. The eroded surfaces of the HEAs, type Ref.+Ti, are shown in Figure 17, Figure 18, Figure 19 and Figure 20. Compared to the stainless steel samples, the HEAs show higher hardness and a multiphase microstructure, which generally makes erosion-induced plastic deformation of HEAs difficult and contributes to minimizing the loss of the erosive mass loss.

In both impingement angles, 30° and 90°, and the reference Ref. (Figure 17) and low titanium addition Ref.+Ti0.05 (Figure 18) HEA samples, the plastic deformation as a result of microcutting is visible, and the increase in titanium addition and therefore hardness affects the combination of microploughing, microcutting, and fatigue spallation, which is observed for Ref.+0.2Ti (Figure 19) and Ref.+Ti0.5 (Figure 20). A higher rate of material deformation is observed for the softer alloys and, consequently, the hardness increases and is well correlated with the erosion resistance of the alloys at both 30° and 90° angles of erosion; see Figure 6 and Figure 13. Much more severe ploughing is observed for samples with lower hardness (Figure 17) while increasing the titanium content results in a microcutting wear mechanism (Figure 20). Therefore, lip formation is a visible result of alumina action, which is also stuck on the eroded surfaces (especially in the case of 90° impingement testing). The sharp edges of the erodent particles not only cut and plough the alloy surfaces but there is also fatigue, resulting in material spallation and even surface cracking (visible in the case of samples Ref.+Ti0.2 and Ref.+Ti0.5 (Figure 19 and Figure 20, respectively)). The more complex samples show less lip formation and a higher action of microcutting is visible.

There is a significant difference in the erosion mechanism for samples of 30° and 90°, and the effect of hardness on the loss of erosion material (see Figure 18) is clearly visible. On the other hand, the hardness derives from the microstructure of the samples. Therefore, combining the size of erosion grooves, scratches, and lips with the size of grains and specific phase dimensions indicates no clear relationship. All HEAs show multiphase microstructures, which, in comparison to single-phase austenitic reference samples, effectively strengthen the HEA erosion resistance and minimize the size of erosion action (shorter and shallower scratches/grooves are noted for HEAs than for stainless steel). The erosion resistance of the solid particles of Ref.+Ti increases with the increasing content of titanium.

## 5. Conclusions

Based on the results shown in the present article, the following conclusions can be formulated:

1. The studied Ref. (Al_0.7_CoCrFeNi) HEA exhibits higher hardness and erosion resistance compared with the state-of-the-art stainless steel AISI 304. As a reason for such a phenomenon, a combination of an HEA multiphase microstructure is claimed.
2. Alloying with Ti results in a further increase in hardness and the erosion resistance of the studied HEA. The resistance to the erosion of solid particles of Al_0.7_CoCrFeNi can be improved by adding titanium Al_0.7_CoCrFeNiTi_x_. The increase in titanium corresponds well to the improvement in the erosion resistance of the Al_0.7_CoCrFeNiTi_x_ alloys.
3. Higher erosion rates were observed for tests conducted at 30 degrees rather than 90 degrees, which is the effect of the dominance of ductile erosion mechanisms.
4. Detailed erosion mechanisms review the grooving, ploughing, and fatigue resulting in the spallation of the material. In the case of the hardest, the surface cracks were noted.
5. As the main reason for the increase in hardness and the stabilization of the resistance to the erosion of BCC, the suppression of α-FCC and the occurrence of Ni_3_Ti phases by Ti are identified. Solid solution and precipitation strengthening mechanisms by the addition of Ti were identified.

## Figures and Tables

**Table 1:** Chemical composition of the studied AISI 304.

Element	Ni	Cr	Fe	Si	Mn	C
Content in wt.%	9.25	18	Bal.	1	2	0.035

**Table 2:** Projected chemical composition of the studied HEA.

Elements	Ni	Co	Cr	Al	Fe	Ti	HEA
Concentration in at. %	21.3	21.3	21.3	14.9	21.3	0.00	Ref.
	21.0	21.0	21.0	14.7	21.0	1.0	Ref.+0.05Ti
	20.4	20.4	20.4	14.3	20.4	4.1	Ref.+0.2Ti
	19.2	19.2	19.2	13.5	19.2	9.6	Ref.+0.5Ti

**Table 3:** Measured chemical composition of the studied HEA.

Elements	Ni	Co	Cr	Al	Fe	Ti	HEA
Concentration in at. %	21.4	21.1	22.1	13.5	21.9	0.0	Ref.
	21.3	19.8	22.5	13.2	21.9	1.3	Ref.+0.05Ti
	20.8	19.1	21.6	12.8	21.3	4.5	Ref.+0.2Ti
	18.6	21.5	19.1	11.8	19.0	10.0	Ref.+0.5Ti

**Table 4:** Measured chemical composition of microstructural components present in the HEA studied as marked in Figure 2a–d.

Point	Element Concentration C, at. %					
	Al	Co	Cr	Fe	Ni	Ti
A	15.0	19.9	23.9	20.7	20.5	0.0
B	11.1	21.4	22.7	22.4	22.4	0.0
C	14.6	18.9	23.1	20.7	21.1	1.5
D	12.2	20.4	22.0	22.3	22.0	1.1
E	9.8	19.9	22.3	22.7	20.7	4.6
F	12.6	18.8	19.6	19.9	21.3	7.9
G	16.3	21.8	14.9	16.1	20.7	10.2
H	6.1	18.4	33.9	25.4	11.2	5.0
I	6.8	22.4	23.9	24.7	16.4	6.0

## Data Availability

The original contributions presented in this study are included in the article. Further inquiries can be directed to the corresponding author.

## Abbreviations

The following abbreviations are used in this manuscript:

HEAs	high entropy alloys
BCC	Body Centered Cubic
FCC	Face Centered Cubic
XRD	X-ray diffraction
SEM	scanning electron microscopy
BSEs	Backscattered Electrons
EDS	Electron Diffraction Spectroscopy
